# P-380. Risk Factors and Clinical Outcomes of Breast Surgery Related Surgical Site Infections in Southeast Michigan

**DOI:** 10.1093/ofid/ofae631.581

**Published:** 2025-01-29

**Authors:** Ambreen Malik, Simran Brar, Anita Shallal, Geehan Suleyman

**Affiliations:** Henry Ford Hospital, Warren, Michigan; Wayne State School of Medicine, Detroit, Michigan; Henry Ford Health, Detroit, Michigan; Henry Ford Health, Detroit, Michigan

## Abstract

**Background:**

Surgical site infections (SSI) after breast surgery are important healthcare-associated infections (HAI) that may delay initiation of chemotherapy for underlying malignancy, in addition to accruing substantial healthcare costs. The reported incidence of breast surgery-related SSI (BSSI) ranges from 0.8%-26% depending on underlying comorbidities. We aimed to describe risk factors and clinical outcomes associated with BSSIs in southeast Michigan.

**Table 1.**
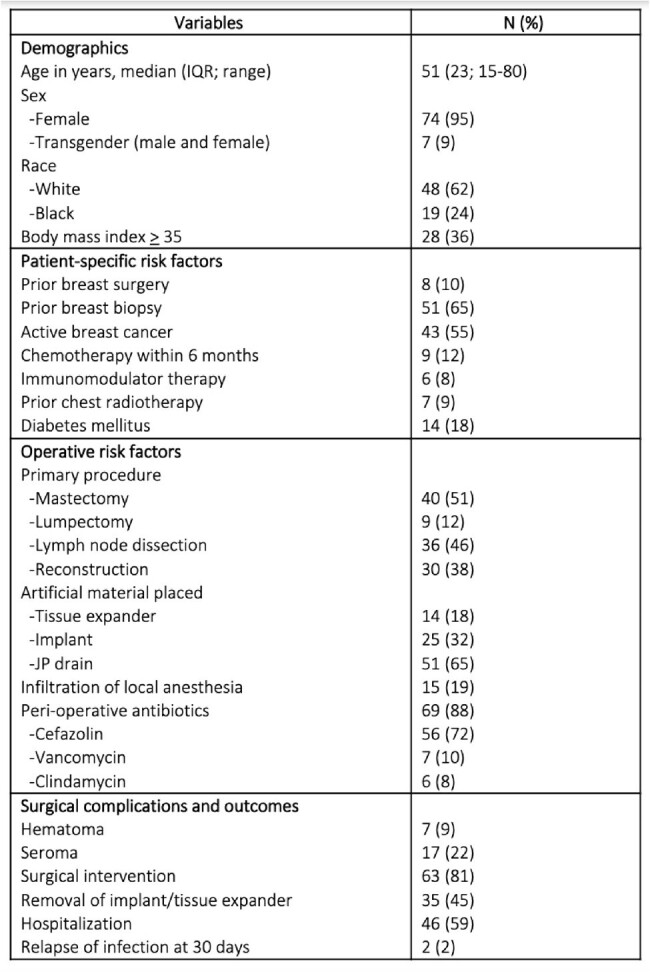
Characteristics of patients with breast surgical site infections

**Methods:**

Retrospective cross-sectional study of adult patients with BSSI at a six-hospital healthcare system. Patients who met the definition of BSSI (per National Healthcare Safety Network) from 2021 to 2023 were included. Charts were reviewed for demographics, comorbidities, risk factors, microbiological data, and clinical outcomes.

**Table 2:**
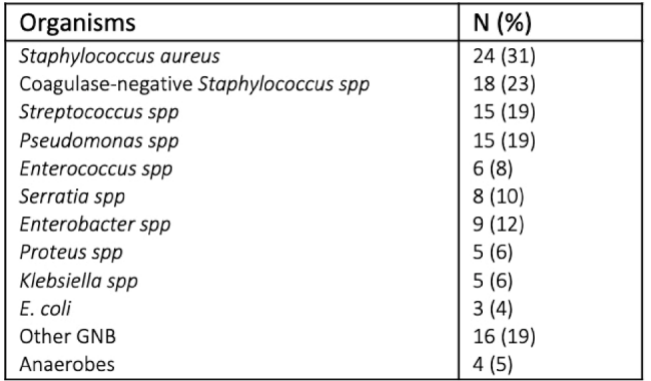
Microbiological data of breast surgical site infections

**Results:**

A total of 146 patients were identified, of which 78 patients (53%) had clinical infection [Table 1]. Most of the patients were female (95%) and white (62%). Common comorbidities included obesity (36%), active breast cancer (55%), and diabetes mellitus (18%). Prior breast surgery (10%), chemotherapy within last 6 months (12%), and prior chest radiotherapy (9%) were less common. Mastectomy was the most common surgery (51%), often with lymph node dissection (46%), and many had artificial material placed (implant; 32% and tissue expanders; 18%). Only 88% of patients received peri-operative antibiotics, of which 72% received cefazolin. The most common microbes isolated were *Staphylococcus aureus* (24%), coagulase-negative *Staphylococcus* spp (23%), *Streptococcus* spp (19%) and *Pseudomonas* spp (19%). Most patients required repeat surgical intervention (81%), with hospitalization (59%) and removal of implants (45%). No patients died at 30 and 90 days after initial surgery.

**Conclusion:**

In this large study, BSSI was often associated with artificial material implantation, and *Staphylococcus aureus* was a common pathogen. Most patients required repeat surgical intervention and removal of implants. Significant opportunities were observed with surgical infection prophylaxis.

**Disclosures:**

**All Authors**: No reported disclosures

